# Two new species of genus *Magadhaideus* Long & Chen, 2017 from China (Hemiptera: Fulgoromorpha: Achilidae)

**DOI:** 10.3897/zookeys.787.26057

**Published:** 2018-10-02

**Authors:** Shi-Yan Xu, Jian-Kun Long, Xiang-Sheng Chen

**Affiliations:** 1 Institute of Entomology, Special Key Laboratory for Development and Utilization of Insect Resources of Guizhou, Guizhou University, Guiyang, Guizhou, 550025, P. R. China Guizhou University Guiyang China; 2 College of Animal Sciences, Guizhou University, Guiyang, Guizhou, 550025, P. R. China Guizhou University Guiyang China

**Keywords:** Achilid, distribution, new species, planthopper, taxonomy

## Abstract

Two new species of the planthopper genus *Magadhaideus* Long & Chen, 2017 from China, *Magadhaideusluchunensis***sp. n.** and *Magadhaideuspingbianensis***sp. n.**, are described and illustrated. Photographs of the new species are provided and a key to species of *Magadhaideus* is also given.

## Introduction

The planthopper tribe Plectoderini (Hemiptera: Achilidae) established by Fennah (Fennah, 1950), containing about 99 genera 346 species, is the largest tribe of Achilidae (Hemiptera: Fulgoromorpha). It is also the most widely disperssed in the world. Plectoderini consists of 13 genera and 26 species (Chen et al. 1989) in China. After that, some new species and genera were described by Wang et al. (2008), Xu and Liang (2012), He et al. (2010), and Long et al. (2015). The planthopper genus *Magadhaideus* (Achilidae: Plectoderini) was established by Long and Chen (2017) with *Magadhaideusxiphos* Long & Chen, 2017 from China as the type species. *Magadhaideus* is similar to *Magadha* in mesonotum bearing a transverse callus on anterior third of disc (readily distinguished from other known genera in the tribe Plectoderini), but differs from *Magadha* in the characteristics of pygofer, medioventral process and phallobase (Long et al. 2017).

The genus, so far, includes only two species, *M.cervina* and *M.xiphos*, which are only found in China (Fennah 1956; Long et al. 2017). In this paper, the specimens of *Magadhaideus*, depositing in the Institute of Entomology, Guizhou University, Guiyang, China, were re-examined, and yielded a further two new species from Yunnan, China. These are described and illustrated, bringing the total number of species of *Magadhaideus* to four species.

## Material and methods

The morphological terminology and measurements used in this study follow Chen et al. (1989) and Yang and Chang (2000). The standard terminology for hind and forewing venation follow Bourgoin et al. (2015). The methods follow Long et al. (2017). Body length was measured from apex of vertex to tip of forewing; vertex length was measured the median length of vertex (from apical transverse carina to tip of basal emargination). The genital segments of the examined specimens were macerated in 10% KOH and drawn from preparations in glycerine jelly using a Leica M125 stereomicroscope. The type material is deposited in the Institute of Entomology, Guizhou University, Guiyang, China (**GUGC**).

## Taxonomy

### Magadhaideus

Taxon classificationAnimaliaHemipteraAchilidae

Long & Chen, 2017 in Long et al. 2017

Magadhaideus Long et al., 2017: 22.

#### Type species.

*Magadhaideusxiphos* Long & Chen, 2017 (original designation).

#### Diagnosis.

Genus diagnostic characters mainly follow Long et al. (2017). Mesonotum with a transverse callus on anterior third of disc (Figs 5, 26; Long et al. 2017: fig. 5); pygofer in lateral view with dorsal margin distinctly shorter than ventral margin (Figs 11, 32; Long et al. 2017: fig. 11); each side of male anal segment with a strong spinous process, directed ventrally (Figs 10, 31; Long et al. 2017: fig. 10); medioventral process of male broad and short, with a small sharp process lateroapically (Figs 12, 33; Long et al. 2017: Fig. 12); phallobase with apical half branched into many more and longer processes.

*Female genitalia.* Anal segment (Figs 17, 38; Long et al. 2017: fig. 17) in dorsal view suborbicular, basal margin M-shaped approximatively. First valvula with five spines (Figs 19, 40; Long et al. 2017: fig. 19). Second valvula with two lateral lobes incompletely symmetrical (Figs 20, 41; Long et al. 2017: fig. 20). Third valvula with outer surface shagreened (Figs 16, 37; Long et al. 2017: figs. 16, 18), in lateral view apical margin sinuate, with an angulate process ventrally (Figs 18, 39; Long et al. 2017: fig. 18).

#### Distribution.

Oriental region (South China).

##### Key to the species of the genus *Magadhaideus* based on males

**Table:** 

1	Medioventral process of pygofer with apical margin truncate, two lateroapical processes small or slender	**2**
–	Medioventral process of pygofer with apical margin not truncate, two lateroapical processes large	**3**
2	Medioventral process of pygofer with apical margin in the middle distinctly convex; phallobase of aedeagus with outermost left lateral processes with apical 1/2 distinctly bent, directed inwards (Fennah, 1956: fig. 15: D)	*** M. cervina ***
–	Medioventral process of pygofer with apical margin in the middle concave; phallobase of aedeagus with outermost left lateral processes with apical 1/2 hardly bent, directed apically (Figs 35–36)	***M.pingbianensis* sp. n.**
3	Medioventral process of pygofer with two slender long lateroapical processes (Long et al., 2017: fig.12); phallobase of aedeagus in ventral	*** M. xiphos ***
–	Medioventral process of pygofer with two much smaller and shorter lateroapical processes, not directed outward (Figure 12); aedeagus with phallobase in ventral view with six processes (Figure 15)	***M.luchunensis* sp. n.**

### Magadhaideus luchunensis sp. n.

Taxon classificationAnimaliaHemipteraAchilidae

http://zoobank.org/3A4A9E53-0871-4563-B5A5-9E9661F001E7

Figs 1–15 , 16–21

#### Type material.

Holotype: ♂, CHINA, **Yunnan**: Lüchun County, Huanglianshan, sweeping, 14 Aug 2014, Zheng-Xiang Zhou. Paratypes: **Yunnan**: 2♂♂5♀♀, same data as holotype; 1♂2♀♀, Lüchun County, Lüboshuiku, sweeping, 13 Aug 2014, Mei-Na Guo; 1♂3♀♀, Lüchun County, Lüboshuiku, sweeping, 8 Aug 2017, Yang-Yang Liu.

#### Diagnosis.

The salient features of the new species include the following: medioventral process of pygofer with two much smaller lateroapical processes (Fig. 12); and phallobase of aedeagus with right basal long lobe single, left apical short lobe directed laterally (Figs 14–15).

#### Description.

Measurements. Body length (from apex of vertex to tip of forewing): male 4.9–5.1 mm (n = 2), female 5.0–5.5 mm (n = 7); forewing length: male 4.6–4.8 mm (n = 2), female 4.7–5.2 mm (n = 7).

#### Colouration.

**Figures 1–15.**
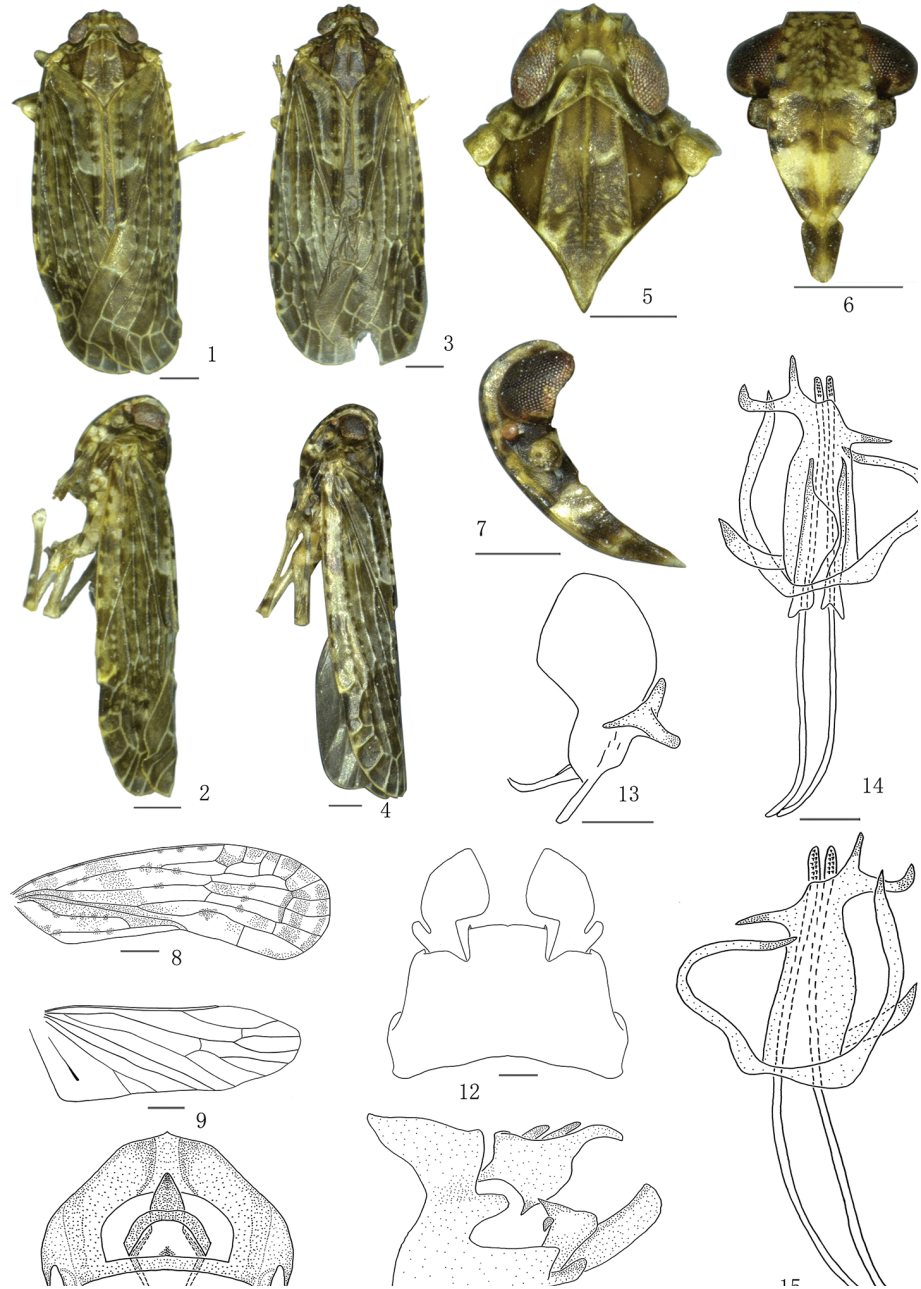
*Magadhaideusluchunensis* sp. n. **1–2** Male habitus (dorsal and lateral views) **3–4** Female habitus (dorsal and lateral views) **5** Head and thorax, dorsal view **6** Face **7** Head, lateral view **8** Forewing **9** Hindwing **10** Anal segment of male, dorsal view **11** Male genitalia, lateral view **12** Male genitalia, ventral view **13** Left genital style, dorsal view **14** Aedeagus, dorsal view **15** Aedeagus, ventral view. Scale bars: 0.2 mm (**8–13**); 0.5 mm (**5–7, 14–15**).

Head yellowish brown. Vertex (Figs 1, 3, 5) along each lateral margin with one dark brown marking at base and another one brown marking at level of anterior margin of eyes, two short longitudinal dark brown markings along median carina apically. Frons (Figure 6) with seven dark brown markings along lateral margin, in middle scattered yellowish-white dots between eyes. Postclypeus pale yellowish, with a transverse brown band near apex. Frontoclypeus (Figure 6) dark brown, with the base and apex pale yellowish. Rostrum yellowish brown, with apex brown. Genae dark brown, as in Figure 7, area along anterior and dorsal margins combined pale yellowish brown, and four transverse short dark brown stripes along anterior margin, a broad transverse yellowish white band under the suture and another narrower one at level of junction of post- and frontclypeus. Eyes (Figs 1–7) generally reddish brown; ocellus (Figs 2, 4, 7) pale reddish. Antennae (Figs 2, 4, 6, 7) pale yellowish brown. Pronotum (Figs 1, 3, 5) brown, lateral lobe with four to five dark brown areas along posterior margin, three carinae pale yellowish brown. Mesonotum (Figs 1, 3, 5) brown, transverse callus pale, posterior two-thirds between lateral carinae with few scattered ivory-white dots, two dark markings at level of lateral carinae and posterior margin joined with peripheries pale yellowish brown, each lateral angle with a large ivory-white marking along posterior margin. Tegula (Figs 1–5) yellowish brown, along posterior margin paler. Forewing (Figs 1–4, 8) greyish brown, veins pale yellowish brown with small variably sized dark markings scattered, clavus with a broad irregular longitudinal dark brown band from base to apex. Hindwing pale brown, veins brown. Legs with colour pattern as in Figs 2 and 4, abdomen dark brown.

#### Head and thorax.

Ratio width of vertex at posterior margin to its length in midline 1.8 (Figure 5), anterior third produced before eyes. Ratio length of frons in midline to its maximum width 1.1, ratio maximum of width to width at apex 1.7. Ratio length of postclypeus in midline to length of frons 0.5 (Figure 6). Rostrum with ratio apical to subapical segment 1.4. Lateral lobes of pronotum with three short longitudinal carinae behind eye, ratio length in midline to length of vertex 0.8 (Figure 5). Mesonotum (Figure 5) in midline 5.3 times longer than pronotum, 2.5 times longer than pronotum and vertex combined. Forewing (Figure 8) with ratio of length to maximum width 3.0, vein R with subapical cell. Hindwing (Figure 9) with length to maximum width ratio of 2.0. Post-tibiae with a lateral spine in basal one-fifths, spinal formula 7–6–6.

#### Male genitalia.

Anal segment in dorsal view (Figure 10) with ratio length to maximum width 1.2, basal margin slightly concave, apical margin angularly convex in middle, in lateral view (Figure 11) with basal 2/3 broad, apical 1/3 slender as finger, roundly bent ventrally, lateral margin near middle with a strong spinous process, directed ventrally. Anal style (Figure 10) not exceeding apical margin of anal segment in lateral view. Pygofer in lateral view (Figure 11) with posterior margin with a large short process in the middle. Medioventral process (Figure 12) with width distinctly wider than length, apical not margin truncate, lateroapical margins with two small sharp processes. Genital style (Figure 13) with apical margin roundly convex, widest part at apical 2/5, a tortuous process rising from basal 1/3 of dorsal margin, branched into three lobes. Aedeagus (Figs 14–15) asymmetrical, phallobase in ventral view (Figure 15) with three long lateral processes rising from base, three short processes rising from apex; in dorsal view (Figure 14) with another two long, irregular, dorsal processes rising from base. Phallic appendages straight with apical margin roundly convex, not exceeding apical margin of phallobase.

#### Female genitalia.

**Figures 16–21.**
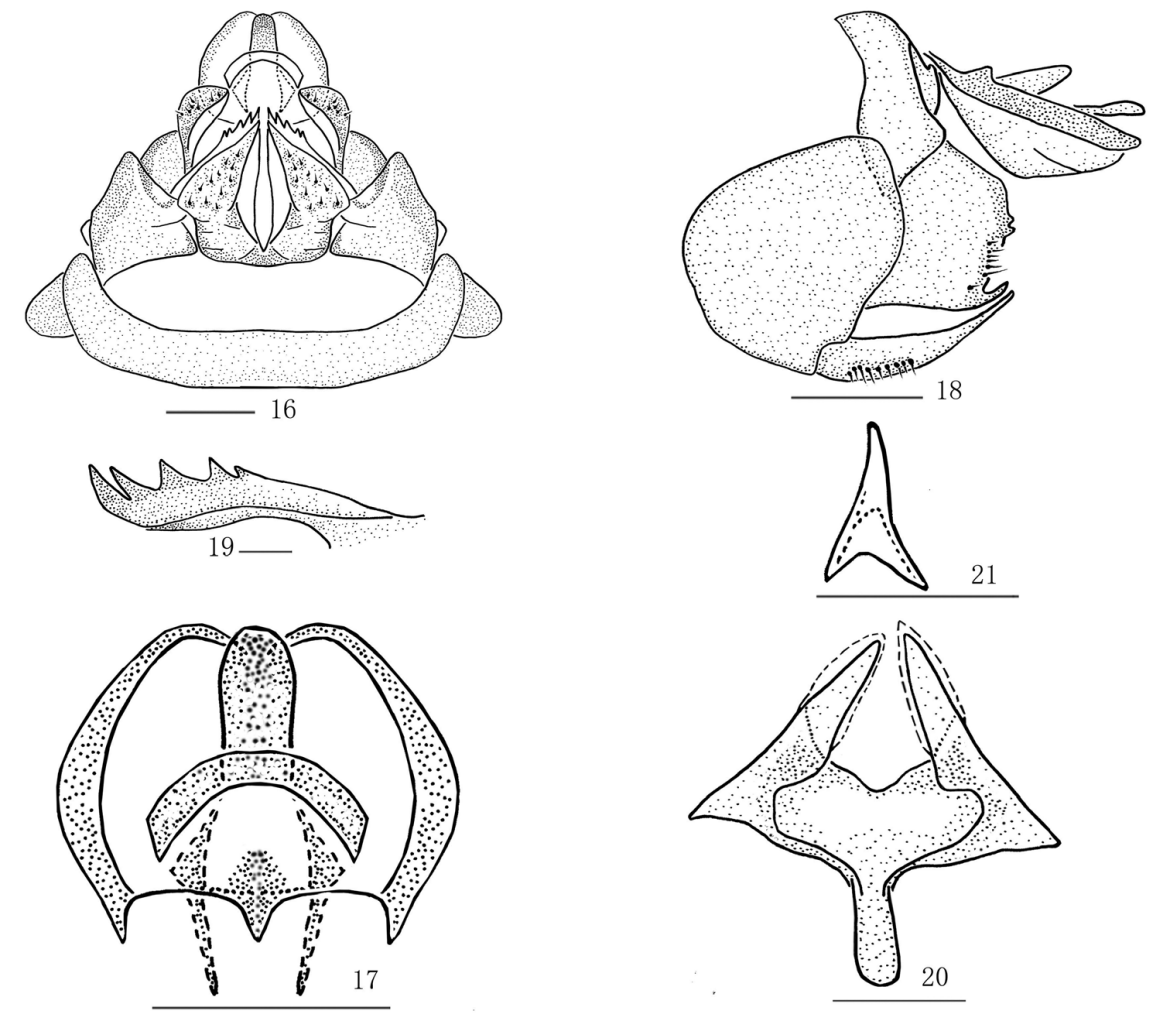
*Magadhaideusluchunensis* sp. n. **16** Female genitalia, ventral view **17** Anal segment of female, dorsal view **18** Female genitalia, lateral view **19** First valvula, from inside **20** Second valvula, ventral view **21** Sclerite on entrance of bursa copulatrix, ventral view. Scale bars: 0.2 mm (**16–18, 20– 21**); 0.5 mm (**19.**)

Seventh abdominal sternum in ventral view (Figure 16), posterior margin truncate or slightly concave. Anal segment (Figs 17–18) in dorsal view with ratio of length to its maximum width 1.4, anal stylet not exceeding apical margin of anal segment. Second valvula in ventral view (Figure 20), with ratio of width to its maximum length 1.1. Sclerite on entrance of bursa copulatrix in ventral view (Figure 21) prominent Y-shaped.

#### Etymology.

This specific name refers to the type locality, Lüchun, Yunnan Province, China.

#### Host plant.

Unknown.

#### Distribution.

China (Yunnan).

#### Remarks.

This species is similar to *M.xiphos* Long & Chen, 2017, but differs from the latter in: medioventral process of pygofer with two small teeth-like lateroapical processes (*M.xiphos* with two finger-like lateroapical processes); phallobase of aedeagus in ventral view with six processes (*M.xiphos* with seven processes).

### Magadhaideus pingbianensis sp. n.

Taxon classificationAnimaliaHemipteraAchilidae

http://zoobank.org/84A1ED6C-97C0-480A-9EC0-2C17C9E2CF33

Figs 22–36 , 37–42

#### Type material.

Holotype: ♂, CHINA: **Yunnan**, Pingbian County, Daweishan, 7 Aug 2014, Zheng-Xiang Zhou; Paratypes: 1♂, as paratypes Qiang Luo, 20 Aug 2017; 4♀♀, 8 Aug 2017, Hai-Yan Sun.

#### Diagnosis.

The salient features of the new species include the following: medioventral process of pygofer with two larger lateroapical processes, directed inward (Figure 33); and phallobase of aedeagus with right basal long lobe branched into two processes, apexes bent, directed inwards, left apical short lobe directed apically (Figs 35–36).

#### Description.

Measurements. Body length (from apex of vertex to tip of forewing): male 4.9– 5.1 mm (n = 2), female 5.0–5.3 mm (n = 4); forewing length: male 3.5–3.9 mm (n = 2), female 4.6–4.8 mm (n = 4).

#### Colouration.

Body with colour pattern (Figs 22–28) except the form of a broad irregular longitudinal dark brown band from base to apex of clavus (Figure 29), as same as *M.luchunensis* sp. n.

**Figures 22–36.**
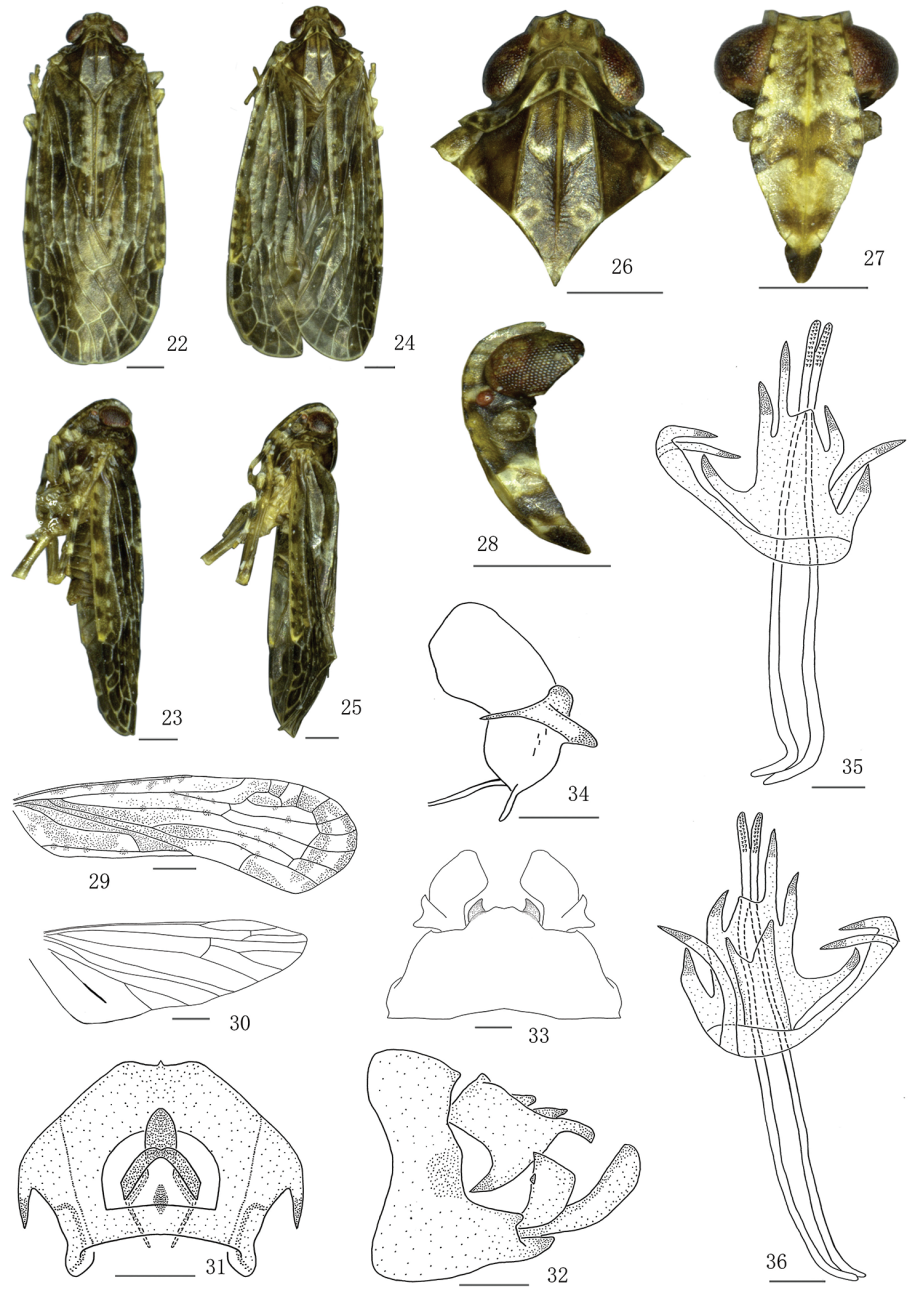
*Magadhaideuspingbianensis* sp. n. **22–23** Male habitus (dorsal and lateral views) **24–25** Female habitus (dorsal and lateral views) **26** Head and thorax, dorsal view **27** Face **28** Head, lateral view **29** Forewing **30** Hindwing **31** Anal segment of male, dorsal view **32** Male genitalia, lateral view **33** Male genitalia, ventral view **34** Left genital style, dorsal view **35** Aedeagus dorsal view, **36** Aedeagus ventral view. Scale bars: 0.2 mm (**29–34**); 0.5 mm (**26–28, 35–36**).

#### Head and thorax.

Ratio width of vertex at posterior margin to its length in midline 2.0 (Figure 26), anterior third produced before eyes. Ratio length of frons in midline to its maximum width 1.2, ratio maximum of width to width at apex 1.8 Ratio length of postclypeus in midline to length of frons 0.5 (Figure 27). Rostrum with ratio apical to subapical segment 1.5. Lateral lobes of pronotum with three short longitudinal carinae behind eye, ratio length in midline to length of vertex 0.8 (Figure 26). Mesonotum (Figure 26) in midline 5.5 times longer than pronotum, 2.5 times longer than pronotum and vertex combined. Forewing (Figure 29) with ratio of length to maximum width 3.0, vein R with subapical cell. Hindwing (Figure 30) with length to maximum width ratio of 2.0. Post-tibiae with a lateral spine in basal 1/5, spinal formula 7–6–6.

#### Male genitalia.

Anal segment in dorsal view (Figure 31) with ratio length to maximum width 1.2, basal margin roundly concave in middle, apical margin angularly convex in middle, anal style not exceeding apical margin of anal segment; in lateral view (Figure 32) with basal 2/3 broad, apical 1/3 slender as finger, roundly bent ventrally, lateral margin near middle with a strong spinous process, directed ventrally. Pygofer in lateral view (Figure 32) with posterior margin slightly sinuate. Medioventral process (Figure 33) short and broad, with two large lateroapical processes, apical margin truncate. Genital style (Figure 34) with apical margin roundly convex, widest part at apical 2/5, a twisted process rising from nearly basal 1/2 of dorsal margin, branched into three lobes. Aedeagus (Figs 35–36) asymmetrical, phallobase in ventral view (Figure 36) with three long lateral processes rising from apex, five long processes rising from base, with another single long irregular flaky bifurcation ventral processes rising from base. Phallic appendages straight with apical margin roundly convex, distinctly exceeding apical margin of phallobase.

#### Female genitalia.

**Figures 37–42.**
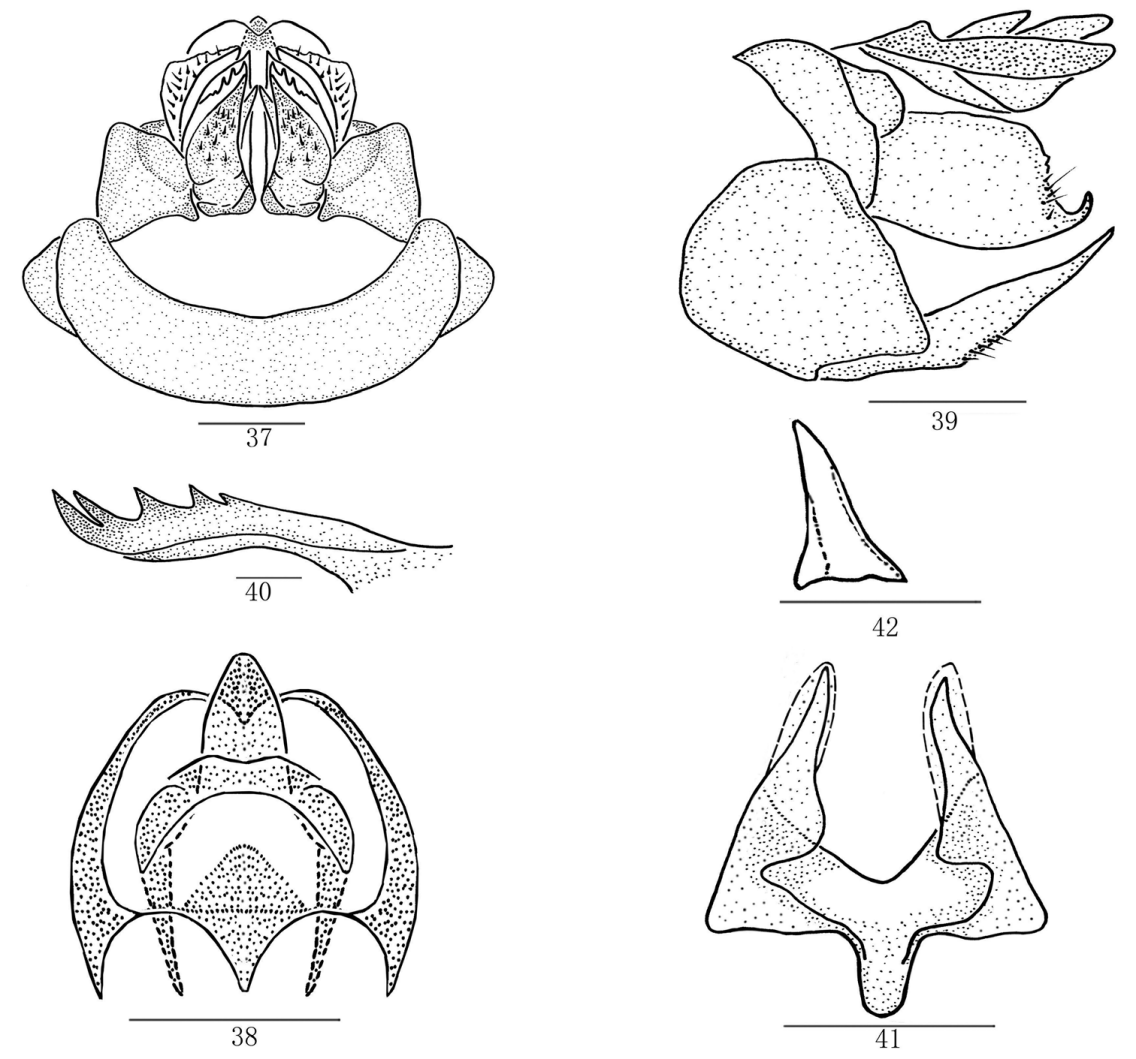
*Magadhaideuspingbianensis* sp. n. **37** Female genitalia, ventral view **38** Anal segment of female, dorsal view **39** Female genitalia, lateral view **40** First valvula, from inside **41** Second valvula, ventral view **42** Sclerite on entrance of bursa copulatrix, ventral view. Scale bars: 0.2 mm (**37–39, 41–42**); 0.5 mm (**40**).

Seventh abdominal sternum in ventral view (Figure 37) with posterior margin distinctly concave. Anal segment (Figs 38–39) in dorsal view with ratio of width to its maximum length 1.0, anal stylet distinctly exceeding apical margin of anal segment. Second valvula in ventral view (Figure 41), with ratio of width to its maximum length 1.2. Sclerite on entrance of bursa copulatrix in ventral view (Figure 42) prominent, T– shaped.

#### Etymology.

This specific name refers to the type locality, Pingbian, Yunnan Province, China.

#### Distribution.

China (Yunnan).

#### Remarks.

This species is similar to *M.cervina* (Fennah, 1956), but differs from the latter in: Medioventral process of pygofer with apical margin in the middle concave (*M.cervina* with apical margin in the middle distinctly convex); phallobase of aedeagus with outermost left lateral processes with apical 1/2 hardly bent, directed apically (*M.cervina* with outermost left lateral processes with apical 1/2 distinctly bent, directed inwards).

## Supplementary Material

XML Treatment for Magadhaideus

XML Treatment for Magadhaideus luchunensis

XML Treatment for Magadhaideus pingbianensis
